# Implication of autophagy in the antifibrogenic effect of Rilpivirine: when more is less

**DOI:** 10.1038/s41419-022-04789-7

**Published:** 2022-04-20

**Authors:** Federico Lucantoni, Ana M. Benedicto, Aleksandra Gruevska, Ángela B. Moragrega, Isabel Fuster-Martínez, Juan V. Esplugues, Ana Blas-García, Nadezda Apostolova

**Affiliations:** 1 Departamento de Farmacología, Facultad de Medicina, Universidad de Valencia, Valencia, Spain; 2 FISABIO–Hospital Universitario Dr. Peset, Valencia, Spain; 3 Centro de Investigación Biomédica en Red de Enfermedades Hepáticas y Digestivas (CIBERehd), Valencia, Spain; 4 Departamento de Fisiología, Facultad de Medicina, Universidad de Valencia, Valencia, Spain

**Keywords:** Macroautophagy, Liver fibrosis

## Abstract

As the main extracellular matrix-producing cells, activated hepatic stellate cells (HSC) are fundamental mediators of liver fibrosis (LF), and understanding their activation/inactivation mechanisms is paramount to the search for novel therapeutics. The antiretroviral drug Rilpivirine (RPV) has demonstrated a hepatoprotective effect in several animal models of chronic liver injury that is related to its antifibrogenic and apoptotic action in HSC. In the present study, we evaluated whether autophagy is implicated in the hepatoprotective action of RPV, as autophagy plays an important role in HSC transdifferentiation. We employed two standard mouse models of chronic liver injury - fatty liver disease and carbon tetrachloride (CCl_4_)-induced hepatotoxicity -and cultured HSC activated with the profibrotic cytokine TGF-β. RPV enhanced autophagy in the whole liver of both mouse models and in activated HSC, evident in the protein expression of autophagy markers, increased autophagosome content and lysosomal mass. Moreover, increased autophagic flux was observed in RPV-exposed HSC as revealed by tandem fluorescence*-*tagged LC3 and p62 and analysis of LC3-II accumulation in cells exposed to the lysosomal inhibitor chloroquine. Importantly, autophagy was involved in the cytotoxic effect of RPV on HSC, though in a differential manner. Pharmacological inhibition of autophagy by 3-methyladenine (3-MA) did not affect the diminishing effect of RPV on viability, while treatment with wortmannin or depletion of specific autophagy proteins (ATG5, Beclin-1 and SQSTM1/p62) rescued the detrimental effect of high concentrations of RPV on the viability of activated HSC. Finally, we also provide evidence that RPV compromises the viability of TGF-β-induced HSC independently of its antifibrogenic effect, observed as reduced collagen 1A1 synthesis, and that this effect does not include RPV´s modulation of autophagy. In summary, as a contributor to the mechanisms involved in the hepatoprotective action of RPV, autophagy may be a good candidate to explore when developing novel therapeutics for LF.

## Introduction

Liver fibrosis (LF) is an essential pathophysiologic consequence of chronic hepatic injury induced by virtually all etiologies including alcoholic steatohepatitis, non-alcoholic fatty liver disease, viral hepatitis, drug-induced hepatitis and others [1, 2]. This reversible wound-healing response is characterized by excessive production of extracellular matrix (ECM), which forms a scar tissue that leads to the disruption of liver parenchyma and enables the progression to cirrhosis, chronic liver failure, portal hypertension and hepatocellular carcinoma. Although removal of the causative agents can lead to a regression of moderate LF (e.g., antiviral therapy in viral hepatitis, or anti-diabetic and lipid-lowering drugs in patients with fatty liver and metabolic syndrome), treatment of advanced LF and cirrhosis is very challenging, and to date there are no specific approved antifibrotic drugs.

A pivotal event in hepatic fibrogenesis is the activation of hepatic stellate cells (HSC); namely, the process of their conversion from quiescent, lipid-storing cells located in the perisinusoidal space to highly proliferating, contractile and migratory myofibroblasts, a phenotype that is considered the main source of ECM during LF [3]. Activated HSC are characterized by their capacity to synthetize ECM components such as fibrillar collagen (types I and III, instead of laminar types IV and VI, which are more common in healthy liver tissue) and to mediate pro-inflammatory actions. Despite the significant effort made during recent years to comprehend the molecular mechanisms involved in HSC activation and de-activation, how exactly these processes occur and how they can be manipulated for therapeutic purposes is still under scrutiny. Multiple studies, both in vivo, using animal models of LF, and in vitro, with primary cultures and immortalized cell lines of HSC, have demonstrated that autophagy plays a role in the activation of HSC [4–7]. Autophagy is a highly regulated catabolic pathway that targets defective or surplus organelles and cellular components to the lysosomes for degradation and is usually stimulated by nutrient restriction, different types of stress, and inflammation [8, 9]. The canonical formation of autophagosomes involves four steps: initiation -which depends on the Unc-51 like autophagy-activating kinase (ULK1) complex-, nucleation -which depends on the Beclin1– phosphatidylinositol 3-kinase class III (PtdIns3KC3)–ATG14L complex and WD-repeat phosphoinositide-interacting proteins (WIPIs), elongation and closure -which depend on ATG12–ATG5 ubiquitin-like and LC3–PE conjugation systems, and recycling -which depends on ATG9. Unlike unconjugated LC3 (LC3-I), which is distributed evenly throughout the cell, the end-product of LC3–PE conjugation (LC3-II) is found on autophagosomes and is interpreted as the bona-fide marker of autophagosome formation [10].

HSC activation is considered a reversible phenomenon, at least to some extent. So far, several processes have been implicated in the resolution of HSC activation including cell death, cellular senescence, and reversion to a quiescent state. How autophagy participates in these processes is still not clear; however, there is evidence that nilotinib and sorafenib, kinase inhibitors employed as antineoplastic drugs, induce apoptosis and autophagic cell death of activated HSC [11, 12].

Recently, we have described that Rilpivirine (RPV), an antiretroviral drug widely employed in the treatment of HIV infection, is hepatoprotective, ameliorating LF in different animal models of chronic liver injury [13]. Specifically, RPV impairs transforming growth factor beta (TGF-β)-activation of cultured HSC, as demonstrated by its negative effect on the expression of general profibrotic markers such as alpha-1 type I collagen (COL1A1), alpha smooth muscle actin (α-SMA), platelet-derived growth factor B (PDGFB), vimentin, plasminogen activator inhibitor-1 (SERPINE1/PAI-1) and TGF-β, and levels of phosphorylated signal transducer and activator of transcription 3(pSTAT3), a transcription factor crucial for HSC survival, proliferation, and activation, therefore contributing to fibrogenesis. In addition, RPV induced apoptosis in HSC through selective STAT1 activation, in a concentration-dependent manner. The aim of the present study is to investigate whether modulation of autophagy is involved in the antifibrogenic effect of RPV in HSC.

## Results

### RPV activates liver autophagy while attenuating liver injury in mouse models of chronic liver disease

**Fig. 1:**
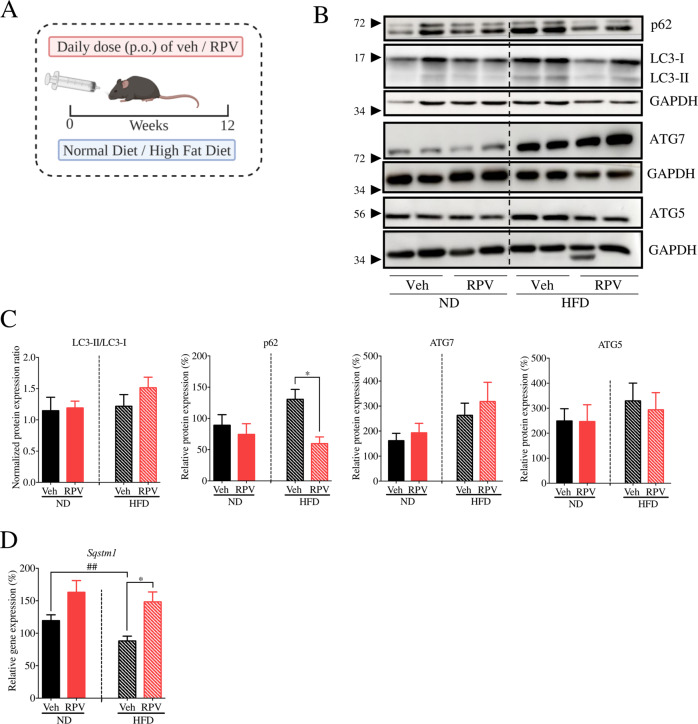
Effect of RPV on hepatic autophagy in a nutritional model of fatty liver disease. **A** Female C57BL/6 J mice received normal diet (ND) or a high-fat diet (HFD) for 12 weeks, and were orally administered (p.o.) either RPV (5 mg/kg/day) or its vehicle (Veh, DMSO). **B** Representative WB images of p62, LC3, ATG5 and ATG7 from total liver protein extracts from 2 mice per group. **C** Ratio of LC3-II/LC3-I levels obtained through densitometric analysis of LC3 signal from WB. Values were normalized to the ratio in the Veh-ND group which was considered 1. Densitometric analysis of p62, ATG5 and ATG7 signal from WB images. Protein expression was normalized with that of GAPDH. **D** Relative gene expression of *Sqstm1* (p62) obtained through RT-qPCR from total liver RNA using *Actb* (actin) as a housekeeping gene. Protein and mRNA levels in the Veh-ND group were considered 100%. Data represented as mean ± SEM (*n* = 8) were statistically analysed by Student´s *t* test of RPV treatment vs Veh (* represents *p* value < 0.05) and HFD vs ND (^##^ represents *p* value < 0.01).

**Fig. 2:**
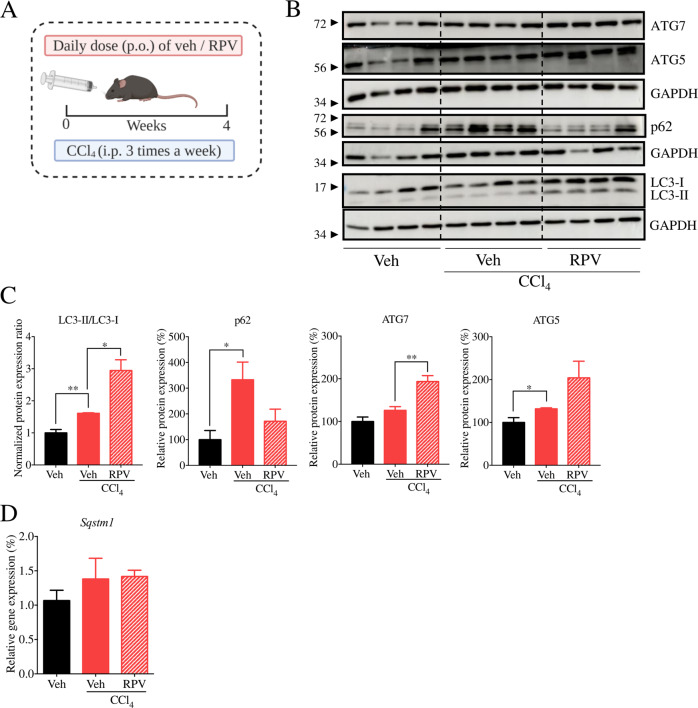
Effect of RPV on hepatic autophagy in a murine model of chronic liver injury induced by a hepatotoxic chemical. **A** Female C57BL/6 J mice were treated for 4 weeks with Veh (DMSO), carbon tetrachloride (CCl_4_)+Veh or CCl_4_ + RPV. CCl_4_ (0.5 mg/kg) dissolved in 50 μL of corn oil was administered intraperitoneally in alternate days while either RPV (5 mg/kg/day) or its vehicle (Veh, DMSO) were orally administered (p.o.). **B** Representative WB images of p62, LC3, ATG5 and ATG7 from total liver protein extracts from 4 mice per group. **C** Ratio of LC3-II/LC3-I levels obtained through densitometric analysis of LC3 signal from WB. Values were normalized to the ratio in the Vehicle group which was considered 1. Densitometric analysis of p62, ATG5 and ATG7 signal from WB images. Protein expression was normalized with that of GAPDH. **D** Relative gene expression of *Sqstm1* (p62) obtained through RT-qPCR from total liver RNA using *Actb* (actin) as a housekeeping gene. Protein and mRNA levels in the Vehicle group were considered 100%. Data represented as mean ± SEM (*n* = 4) were statistically analysed by Student´s *t* test of CCl_4_-RPV treatment vs CC_4_-Veh and CC_4_-Veh vs Veh (* and ** represent *p* value < 0.05 and < 0.01 respectively).

We have previously described that RPV is hepatoprotective in mouse models of chronic liver injury including non-alcoholic fatty liver disease (NAFLD) and carbon tetrachloride (CCl_4_)-induced hepatotoxicity [13], and in the present study, we determined whether these effects involve an impact on hepatic autophagy. To this end, for the NAFLD model, mice were divided into 4 groups—normal diet (ND) + Veh, high fat diet (HFD) + Veh, ND + RPV and HFD + RPV (Fig. 1A). WB analysis of whole liver tissue revealed that, while HFD did not alter LC3II/I ratio, it provoked an increase in the levels of classical autophagy markers ATG5 and ATG7. HFD also enhanced the protein content of SQSTM1/p62 compared to ND (Fig. 1B, C) while RT-qPCR analysis showed a diminished *Sqstm1* mRNA levels in HFD mice (Fig. 1D). These findings point to altered autophagy in the liver of HFD mice. Treatment with RPV in HFD-exposed animals slightly incremented the LC3II/I ratio and ATG7 levels while producing a major decrease in SQSTM1/p62 protein levels, effects indicative of enhanced autophagy (Fig. 1B, C). Notably, the diminishing effect on p62 was not due to a decrease in *Sqstm1* expression, as RPV actually augmented *Sqstm1* mRNA in both ND- and HFD-exposed animals (Fig. 1D). Altogether, these data point to the capacity of RPV to enhance autophagy in the livers of HFD-exposed animals. In the CCl_4_ model, mice were divided in 3 groups: Control (Veh), CCl_4_-exposed+Veh and CCl_4_ + RPV (Fig. 2A). Whole-liver analysis of the protein levels of several autophagy markers (increase in LC3II/I ratio, enhanced expression of ATG5 and ATG7, and diminished levels of p62) revealed that RPV seems to enhance autophagy in CCl_4_-exposed mice (Fig. 2B, C). While treatment with CCl_4_ slightly upregulated the mRNA levels of *Sqstm1*, RPV did not modify this effect (Fig. 2D). Altogether, RPV enhances the expression of markers of autophagy in the livers of mice challenged with CCl_4_.

### RPV enhances autophagy in hepatic stellate cells

**Fig. 3:**
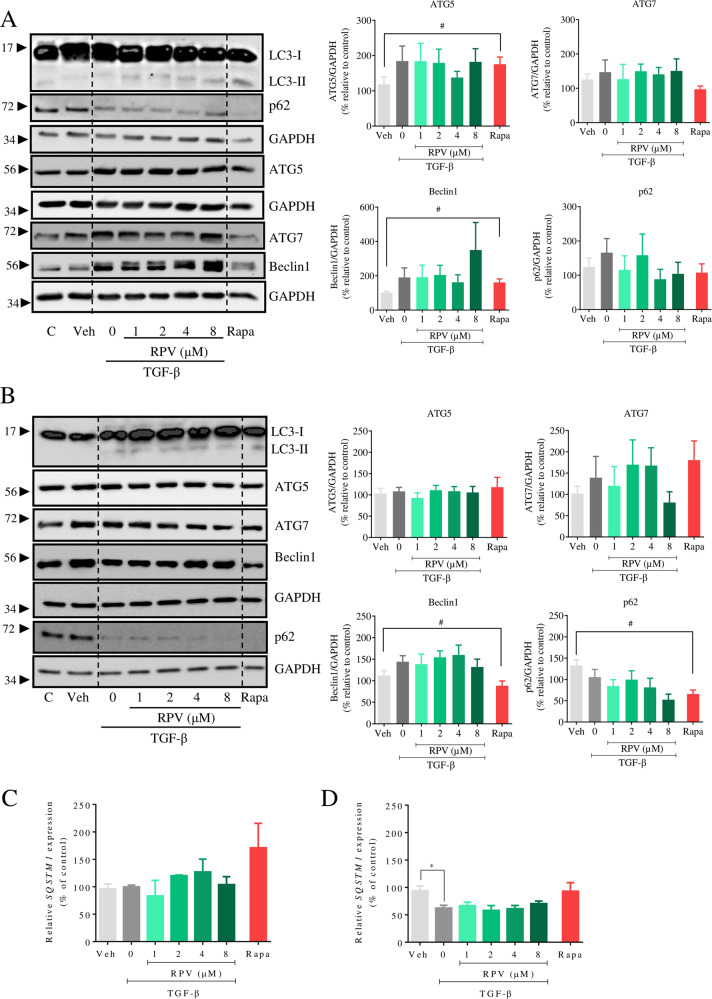
Analysis of autophagic markers in activated LX-2 cells treated with RPV. LX-2 cells were exposed to vehicle, TGF-β or TGF-β plus RPV (1, 2, 4, and 8 µM) for 24 h or 48 h. Representative images of immunoblots for LC3, p62, ATG5, ATG7, and Beclin-1 using whole cell protein extracts of cells treated for 24 h (**A**) and 48 h (**B**) and summary of densitometry data of treatments. Expression was normalized with that of GAPDH as a reference protein. Relative gene expression of *SQSTM1* (p62) obtained by RT-qPCR of cells treated for 24 h (**C**) and 48 h (**D**). Data which represent mean ± SEM (*n* = 3–6) are expressed in relation to those of untreated cells in each experiment, which was considered 100%. Statistical analysis was performed with one-way ANOVA with Bonferroni post-test for RPV + TGF-β versus TGF-β treatment or with paired Student´s *t* test for TGF-β vs Vehicle (* represents *p* value < 0.05) and Rapamycin (Rapa) vs Vehicle (^#^ represents *p* value < 0.05).

**Fig. 4:**
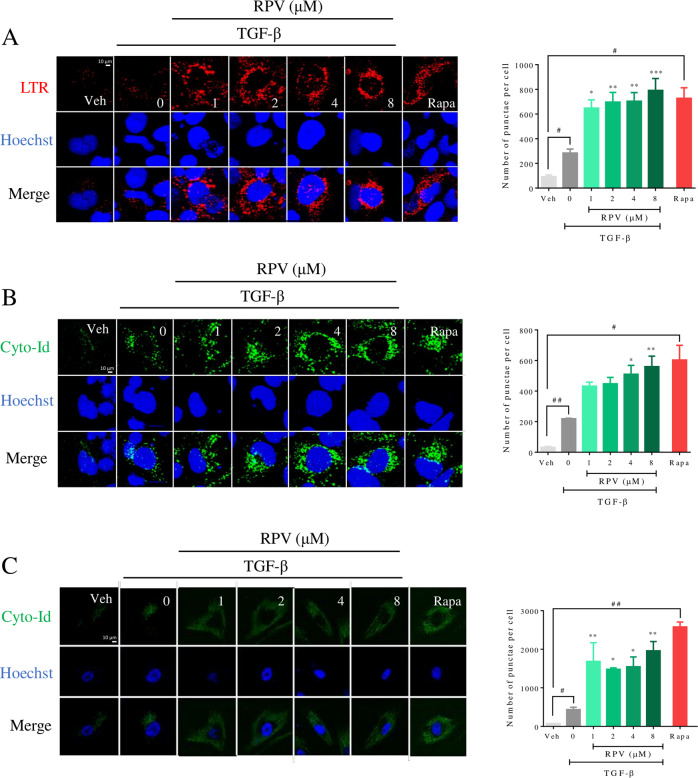
RPV induces the accumulation of lysosomes and autophagic structures in activated HSC. LX-2 or primary human HSC were treated with vehicle, TGF-β or TGF-β plus RPV (1, 2, 4, and 8 µM) for 48 h. **A** LX-2 cells were stained with Lysotracker (LTR, lysosomes) and Hoechst 33342 (nuclei) and live cells fluorescence images were taken with a confocal microscope. Representative images and quantification of LTR signal are shown. **B** LX-2 cells were stained with Cyto-Id (autophagic structures) and Hoechst 33342 (nuclei) and live cells fluorescence images were taken with a confocal microscope. Representative images and quantification of Cyto-Id signal are shown. **C** Representative images of Cyto-Id and Hoechst 33342 and quantification of Cyto-Id signal in primary human HSC. All images were analysed with a custom made CellProfiler pipeline to obtain the median number of punctae per cell. Bars represent mean ± SEM (*n* = 3). Data were analyzed by one-way ANOVA with Bonferroni post-test for RPV + TGF-β v TGF-β treatment (*, ** and *** represent a *p* value < 0.05, < 0.01 and < 0.001 respectively), or paired Student *t* test of TGF-β vs Vehicle and rapamycin (Rapa) vs Vehicle (^#^ and ^##^ represent a *p* value < 0.05 and < 0.01, respectively).

The hepatoprotective action of RPV in vivo occurs with diminished fibrogenic activation of HSC in parallel to the induction of apoptosis in these cells [13]. In the present study, we sought to analyse whether autophagy modulation is part of the effect of RPV on HSC during their activation, for which LX-2 cells treated with the profibrotic cytokine TGF-β (24 h or 48 h) were co-exposed to clinically relevant concentrations of RPV. TGF-β signaling is considered the key fibrogenic pathway that drives HSC activation and induces ECM production [14]. During acute and chronic liver injury, TGF-β is activated from deposits in the ECM and also released from various cell types. HSC are considered both a pivotal source and target of TGF-β. Firstly, several classical markers of autophagy were assessed by WB, including LC3, Beclin-1, ATG5, ATG7 and p62 [10]. Treatment with TGF-β has been described to induce autophagy in LX-2 cells [15]. In our study, it resulted in a tendency to increase levels of ATG5 and Beclin-1 after 24 h (Fig. 3A) of treatment, which tended to normalize at 48 h (Fig. 3B). An increased LC3II/LC3I ratio and a diminished level of p62, both indicative of enhanced autophagy, were observed with TGF-β, especially at 48 h (Fig. 3B). Of note, co-exposure to TGF-β and 8 μM RPV further enhanced the LC3II/I ratio and Beclin-1 at 24 h, while inducing a concentration-dependent drop of p62/SQSTM1 levels at 48 h. In order to assess whether this decrease in p62 was due to changes in its mRNA level, we performed RT-qPCR analysis of *SQSTM1* expression. This experiment revealed that treatment with TGF-β significantly diminished *SQSTM1* mRNA levels at 48 h, with no effect at 24 h (Fig. 3C, D). RPV did not modify *SQSTM1* mRNA in TGF-β-treated cells at either 24 h or 48 h. Enhancement of autophagy usually produces an increase in the lysosomal content. We sought to evaluate this effect in our model by live single-cell microscopy using the fluorochrome Lysotracker Red (LTR) and noted that treatment with RPV (48 h) led to a major increase in the lysosomal signal, which was also observed with the positive control of auptohagy induction, rapamycin (Fig. 4A). Increased autophagosomal content was also displayed by Cyto-Id. As expected, in this experiment TGF-β-treatment of LX-2 cells (48 h) incremented the fluorescent signal emitted by autophagic vacuoles, and this effect was further enhanced by co-treatment with RPV in a concentration-dependent manner (Fig. 4B). In order to verify that the results of enhanced autophagy by RPV were not an artifact of the immortalization process during the generation of LX-2 cell line, we reproduced this experiment in primary human HSC, obtaining a similar result (Fig. 4C). Of note, in these quantitative evaluations (Fig. 4), the results with RPV resembled those obtained with the classical autophagy inducer rapamycin.

**Fig. 5:**
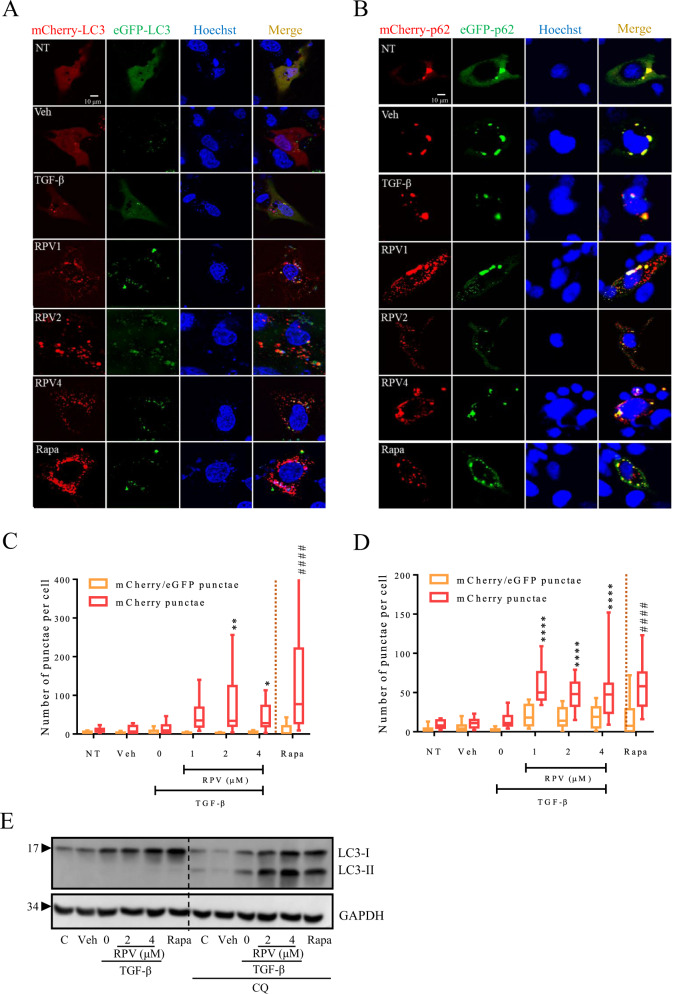
RPV induces accumulation of p62 and LC3 in late autophagic structures. Representative confocal microscopy images of LX-2 cells that express mCherry-eGFP-LC3 (**A**) or mCherry-GFP-p62 (**B**) and had been treated with vehicle, TGF-β or TGF-β plus RPV (1, 2, and 4 µM) for 24 h. Cells were stained with Hoechst to detect nuclei. Red signal indicates the presence of the protein of interest in late autophagic structures (autophagolysosomes), while yellow signal (red plus green fluorescence) indicates its presence in early autophagic structures (autophagosomes). **C**, **D** Quantification of the fluorescence signal data. Images were analyzed with a custom made CellProfiler pipeline to detect red (red boxplots) and green fluorescent signal and to relate the two fluorescent signals (red/green double positive—orange boxplots). Bars represent median ± interquartile range (IQR, *n* = 3); all values were analyzed by two-way ANOVA with Bonferroni post-test for RPV + TGF-β vs TGF-β treatment (*, ** and **** represent a *p* value < 0.05, < 0.01 and < 0.0001 respectively) or paired Student´s *t* test of TGF-β vs vehicle and rapamycin (Rapa) vs vehicle (^#####^ represents a *p* value < 0.0001). **E** Analysis of the autophagic flux in LX-2 cells treated with vehicle, TGF-β or TGF-β plus RPV (2 and 4 µM) for 48 h under conditions of normal autophagy or those of inhibited autophagosome-lysosome fusion (cotreatment with chloroquine (CQ)). WB images of LC3 and GAPDH as a reference protein are representative of three independent experiments. Rapamycin (Rapa) was used a positive control of autophagy induction.

In addition, the autophagosome maturation process and autophagic flux can also be monitored using a tandem fluorescence-tagged LC3 such as mCherry-GFP which enables to distinguish autophagosomes (yellow fluorescence) from autophagolysosomes (red fluorescence). Using this system, we showed that 24h-treatment with RPV increased the number of LC3-containing autophagosomes and autolysosomes in a similar way to the positive control for autophagy induction, rapamycin (Fig. 5A, C). A similar result was obtained when cells expressed mCherry-eGFP-p62 (Fig. 5B, D). Autophagic flux was also assessed by studying LC3 levels in cells in which autophagy was blocked with chloroquine (CQ), an inhibitor of the lysosome-autophagosome fusion. These experiments revealed an increased accumulation of LC3-II in TGF-β-activated LX-2 after 48 h of co-treatment with RPV and CQ compared to treatment with RPV only (Fig. 5E). Altogether, these data strongly suggest that RPV enhances autophagy in TGF-β-treated HSC.

### Inhibition of autophagy differentially affects the action of RPV in hepatic stellate cells

**Fig. 6:**
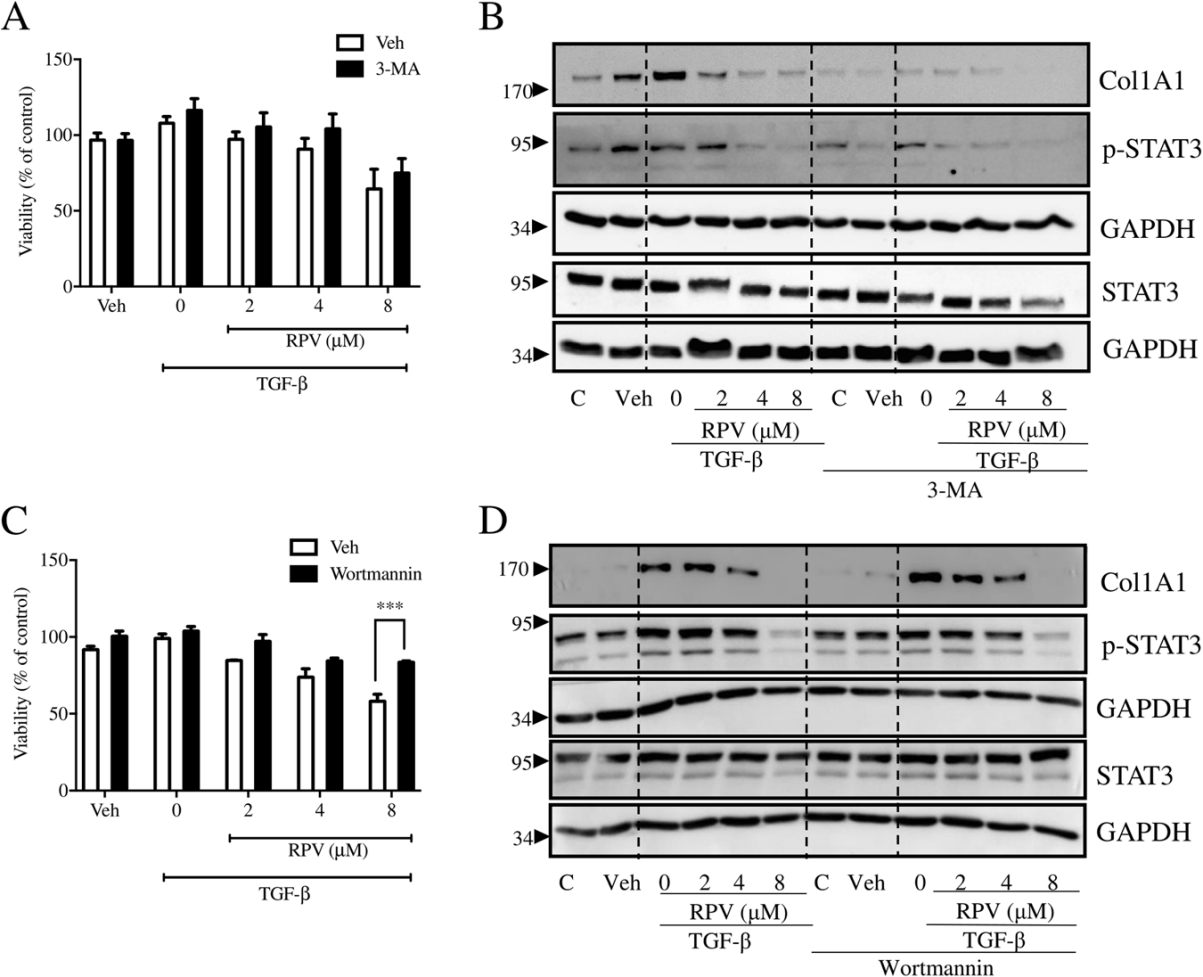
Co-treatment of RPV with the autophagic inhibitors 3-MA and wortmannin shows a differential effect between the two. Unlike wortmannin which lacked an effect, 3-MA diminished the content of collagen 1A1 produced by activated LX-2 cells. 3-MA did not affect RPV-induced decrease in cell viability while wortmannin rescued it. Viability of LX-2 cells treated with vehicle, TGF-β or TGF-β plus different concentrations of RPV in the presence or absence of 3-MA (**A**) or wortmannin (**C**) was determined with acid phosphatase assay. Bars represent mean ± SEM (*n* = 5 for 3-MA and *n* = 3 for wortmannin); data are expressed as in relation to that of untreated control cells in each experiment for both with and without 3-MA or wortmannin, which was considered 100%. Statistical analysis was by two-way ANOVA with Bonferroni post-test of 3-MA or wortmannin treatment vs vehicle (*** represents a *p* value < 0.001). **B**, **D** Representative WB images of Col1A1, p-STAT3, STAT3, and GAPDH in LX-2 cells exposed to vehicle, TGF-β or TGF-β plus RPV (2, 4, and 8 μM) for 48 h in the presence or not of 3-MA (*n* = 3) or wortmannin (*n* = 3).

**Fig. 7:**
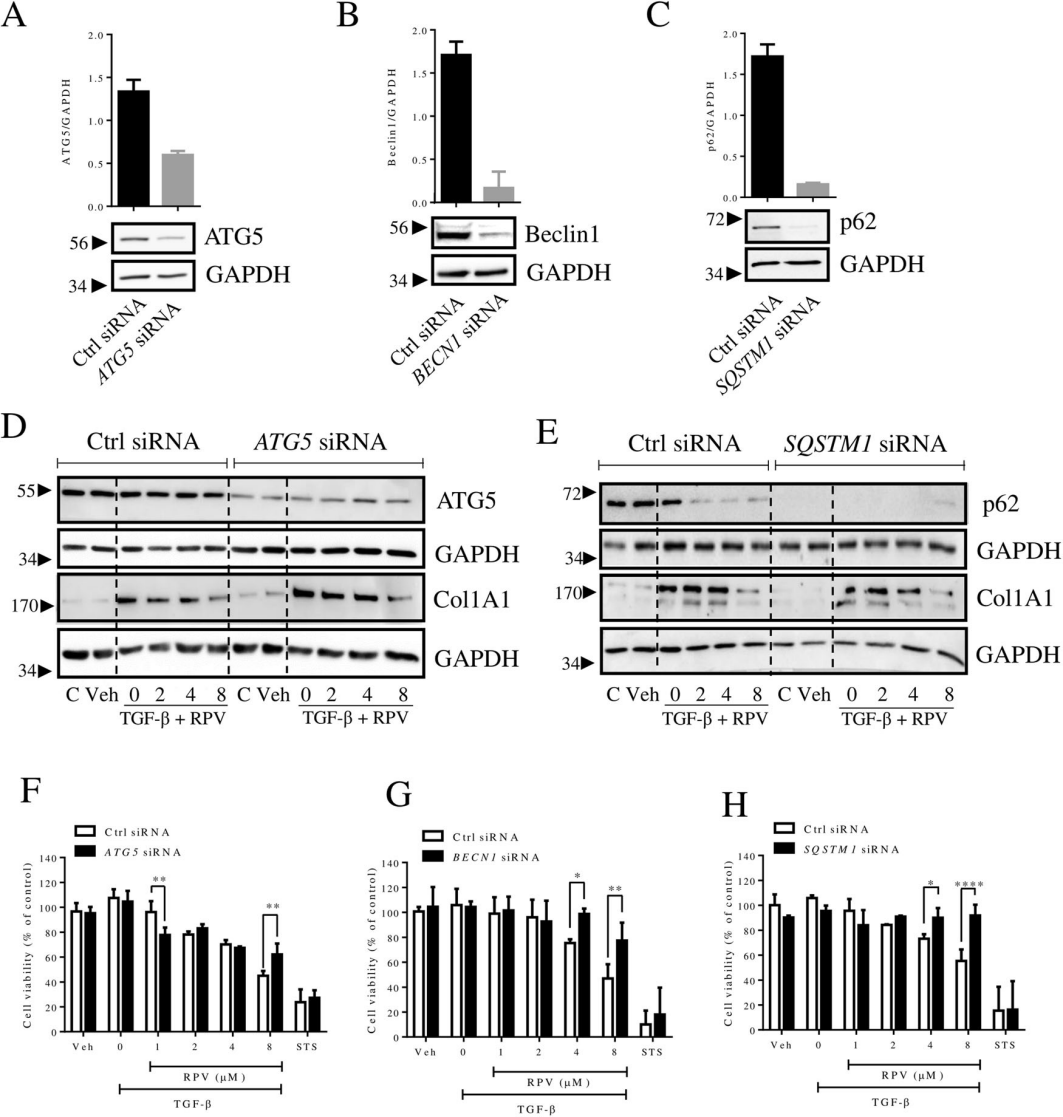
RPV’s effect on cell viability is partially rescued by silencing proteins involved in autophagy. Specific autophagy genes were silenced by siRNA in LX-2 cells (24 h) which were then treated with TGF-β or TGF-β plus different concentrations of RPV for 48 h. Representative WB images and quantification of the protein levels of autophagy related genes—(**A**) *SQSTM1*, (**B**) *ATG5* and (**C**) *BECN1* following transfection with siRNA (**D**) Representative WB images of ATG5 and collagen 1A1 in control or *ATG5*-silenced cells. **E** Representative WB images of p62 and collagen 1A1 in control or *SQSTM1*-silenced cells. GAPDH was employed as a loading control. Viability of cells exposed to control siRNA vs (**F**) *ATG5*, (**G**) *BECN1* and (**H**) *SQSTM1* silenced cells treated either with TGF-β or different concentrations of RPV plus TGF-β for 48 h. Cell viability was assessed using acid phosphatase assay. All data represent mean ± SEM (*n* = 3) and are expressed as in relation to those of untreated cells in each experiment, which was considered 100% (both in control siRNA and in gene-specific siRNA). Statistical analysis was by two-way ANOVA with Bonferroni post-test of control siRNA vs gene-specific siRNA (*, ** and **** represent a *p* value < 0.05, < 0.01 and < 0.0001, respectively).

In order to analyze whether modulation of autophagy participates in the antifibrogenic and pro-apoptotic effect of RPV in HSC, the drug’s action was assessed in cells in which autophagy was inhibited either pharmacologically or through silencing of genes involved in autophagy. As we reported previously [13], treatment with RPV concentration-dependently compromises cell viability in HSC, and this effect was corroborated in the present experiments (48 h exposure of LX-2 cells), as shown in Fig. 6A. Cells treated with 3-MA, a widely used autophagic inhibitor that interferes with the early stages of autophagosome initiation through its inhibitory action on class III phosphatidylinositol 3 kinase (PI3K), displayed diminished viability (Supplementary Fig. 2). Interestingly, the effect of RPV on cell viability was present in cells co-treated with 3-MA, to a similar level as in cells with functional autophagy. Moreover, we observed that autophagy was necessary for the fibrogenic activation of LX-2 cells under TGF-β treatment, as seen by the lack of collagen 1A1 expression in 3-MA-treated cells (Fig. 6B). Despite its wide use as an up-stream autophagy inhibitor, 3-MA can exert autophagy-independent actions including an antifibrotic effect through interference with the binding of Smad3 and p300 [16]. In addition, inhibition of PI3K can negatively affect pro-survival signaling pathways such as AKT and protein synthesis, and 3-MA can also induce cell death independently of its effect on PI3K [17]. For these reasons, we also repeated the experiment using another autophagy inhibitor, wortmannin, an irreversible inhibitor of PI3K and observed that co-treatment with wortmannin rescued the effect of RPV on cell viability (Fig. 6C). Interestingly, wortmannin´s action on fibrogenesis was different from that of 3-MA as it lacked effect on RPV-induced decrease of collagen 1A1 synthesis or STAT3 activation upon TGF-β exposure (Fig. 6D). These findings showed that despite being both early-stage autophagy inhibitors and thus often used interchangeably, 3-MA and wortmannin can have differential effects in certain settings. Considering that conclusions based solely on pharmacological regulation of autophagy can be misleading, we also inhibited autophagy by silencing *ATG5, SQSTM1* and *BECN1*. ATG5, part of the ATG12-ATG5 conjugation system, mediates phagophore expansion and autophagosome formation, but is not required for the initiation of autophagy. Cells transfected with *ATG5*-silencing siRNA displayed 44% of the ATG5 level compared to cells transfected with Control siRNA (100%), as can observed in Fig. 7A, and exhibited lower cell viability compared to cells with control transfection (Supplementary Fig. 2). Nevertheless, cells with diminished ATG5 content were able to produce collagen 1A1 upon TGF-β treatment at similar levels to those fully expressing ATG5 (Fig. 7D). In addition, co-treated cells (TGF-β + RPV) with diminished ATG5 content displayed the same levels of collagen 1A1 as co-treated cells with normal ATG5 (Fig. 7D). Very similar results were obtained in cells in which SQSTM1/p62, a classical receptor of autophagy, had been almost entirely depleted (Fig. 7C and E and Supplementary Fig. 2). These observations suggest that the fibrogenic process stimulated with TGF-β and, alongside it, the antifibrotic effect of RPV under these stimulatory conditions, occur in an ATG5- and p62-independent manner.

Next, we analyzed whether depletion of the aforementioned key autophagic proteins (by means of RNA interference) affected the compromising effect of RPV on cell viability in LX-2 cells. Exposure of si*ATG5*-transfected cells to RPV revealed a differential effect that varied according to the concentration. Lower concentrations (1 μM) of RPV that did not affect or only slightly diminished the LX-2 viability of cells with normal ATG5 levels had the same effect, or showed a slightly more detrimental effect, on the viability of cells with severely diminished ATG5. The contrary was observed in cells treated with a higher concentration of RPV (8 μM), which compromised cell viability when ATG5 was present but had no effect in cells that displayed lower ATG5 (Fig. 7F). A very similar observation was made regarding cell viability in cells in which *BECN1* (Fig. 7G) or *SQSTM1* (Fig. 7H) was silenced. In these two cases, the detrimental effect of RPV on cell viability was abolished even at 4 μM RPV. This could have been related to the fact that, while *BECN1* and *SQSTM1* were silenced effectively (90% reduction), ATG5 was reduced by only 55%. Importantly, depletion of any one of the three proteins had no effect on the cytotoxic action of staurosporine (STS), which was employed here as an inducer of apoptotic cell death, which occurs without participation of autophagy.

Altogether, these data indicate that autophagy plays a role in the cytotoxic effect of RPV on TGF-β-stimulated LX-2 cells. However, the effect is complex as it occurs independently of RPV´s capacity to diminish collagen 1A1 content which appears to be unrelated to autophagy.

## Discussion

The present study provides evidence that autophagy is involved in the previously described hepatoprotective and antifibrogenic actions of RPV both in vivo and in vitro. We show that clinically relevant concentrations of this drug induce autophagy in two mouse models of liver injury, HFD-induced model of NAFLD and fibrosis induced by the hepatotoxin CCl_4_. Abundant evidence supports that hepatic autophagy is diminished in NAFLD in both HFD-induced mouse models [18–20] and in human samples [18, 21]. In the present study, autophagy in whole-liver samples of NAFLD mice was altered but the available evidence did not point necessarily to diminished autophagy. Of note, RPV enhanced autophagy in the livers of our HFD-exposed animals. A similar effect was observed in the livers of RPV-treated mice exposed to CCl_4_. Nevertheless, studies using whole-liver samples do not reflect the effect in the different cell types and given that the majority of liver cells are hepatocytes, the observed phenomenon may reflect the effect in this cell type. Also, in order to specifically pinpoint the role of HSC autophagy in the protective effect of RPV in vivo, it is paramount to perform experiments in animals in which autophagy had been suppressed selectively in HSC (by using genetic approaches).

Numerous studies, both in vitro and in vivo, have described the participation of autophagy in HSC activation [22], but how this process is regulated and how exactly it interferes with HSC transdifferentiation is still a matter of debate. Importantly, in contradiction of the established view of autophagy being profibrogenic in HSC, there is mounting evidence supporting the antifibrotic role of autophagy in these cells [23, 24]. Specifically, drug-induced excessive autophagy has been reported to induce cell death in activated HSC [25, 26]. Alternatively, autophagy may regulate the levels of the main profibrotic mediators, including collagens [27] or profibrogenic exosomes [28]. Here we describe that RPV enhances autophagy in TGF-β-activated HSC and increased autophagy seems to be involved in the capacity of the drug to induce death in these cells. This is relevant given the fact that, among other proposed HSC-targeted strategies, such as inducing senescence or reversal (deactivation), enhancing apoptosis in activated HSC is considered a promising approach to treat LF [29]. Our study shows that 3-MA is antifibrogenic, which is in line with several previous studies with CCl_4_- or ethanol-induced LF in mice [7, 30] and cellular models including LX-2 cells activated by TGF-β [31] or As_2_O_3_ [32]. Similar observations have been made in other cell types, such as normal human dermal fibroblasts (NHDFs) [16] or mesangial cells (MC), the fibrogenic cell type in the kidney, in which treatment with 3-MA inhibited collagen 1 synthesis [4]. Actually, 3-MA was reported to diminish renal fibrosis by abolishing TGF-β among other effects [33]. The fact that RPV diminished cell viability equally in the presence and in the absence of 3-MA may suggest that inhibition of autophagy in its early stages has no impact on the cytotoxic effect of RPV. However, this result was not corroborated with wortmannin, another early-stage autophagy blocker and also a PI3K inhibitor, which did not affect RPV´s action on TGF-β-induced collagen 1A1 synthesis while rescuing RPV´s effect on cell viability. The explanation for these differences may reside in the different targets of 3-MA and wortmannin. In comparison with wortmannin which inhibits class III PtdIns3K, 3-MA has been shown to inhibit class III PI3K only transiently and instead it is a persistent inhibitor of class I PI3K [34]. There is evidence that PI3K signaling plays a role in TGB-β induced collagen 1 synthesis. For example, in human MC, TGF-β1-induced collagen 1 gene expression is not only Smad3-dependent but TGF-β1 also activates PDK-1 and Akt, two downstream targets of PI3K. Inhibition of the PI3K pathway with LY294002 or an Akt dominant-negative construct diminishes Smad3 activity and abrogates TGF-β1-stimulated collagen gene transcription [35]. The results of wortmannin in LX-2 cells in the present work are similar to those obtained with deprivation of ATG5, p62 or Beclin-1. Therefore, alteration of autophagy would appear to be behind the effect of RPV on HSC survival. Interestingly, with all three proteins, we observed a differential effect that depended on the concentration of RPV. Depletion of these proteins had either no effect or aggravated RPV´s action on cell viability when RPV was applied at lower concentrations, whereas the opposite was observed when the drug was present at higher concentrations (cell viability was partially recovered). Therefore, it is not only autophagy as a process, but its levels of activation that account for the cytotoxic action of RPV in HSC. While moderate stimulation of autophagy by RPV does not produce a major effect on cell viability, and can even be protective, massive induction of autophagosome generation, like that produced with high concentrations of RPV, promotes cell death.

Of note, our findings point to an important fact: fibrogenesis and cell proliferation/cell death of HSC are regulated independently. We show that collagen 1A1 levels do not necessarily correlate with cell viability as cells with only a slight decrease in viability or even increased viability (as in the case of TGF-β + 3-MA) were virtually void of collagen 1A1. This is highly relevant and points to the fact that death of HSC and the reversal of their activation should be regarded as independent processes, at least under certain conditions. Also, while the effect of RPV regarding cell viability seems to depend on autophagy, RPV´s action on TGF-β-induced collagen 1A1 synthesis is autophagy-independent.

The present study offers two possible explanations for the antifibrotic effect of RPV. On the one hand, RPV enhances the process of TGF-β-triggered autophagy, and this over-stimulation exerts detrimental effects on cell viability. This effect is independent of RPV´s ability to diminish collagen 1A1 levels. On the other hand, autophagy is a redundant process and not always relies on canonical regulators (such as ATG5 or ATG7) [36–38]. Moreover, many of the components of autophagic machinery have been described to have autophagy-independent roles. ATG5, ATG7, p62 and Beclin-1, among others, are reported to be involved in a form of regulated cell death mediated by autophagy-relevant proteins or “autophagy-dependent cell death” (ADCD) according to recent nomenclature guidelines. In mammals, this stands out as an autophagy-independent pathway under the control of the autophagic machinery [39]. Whether this is the case in the present model requires further research.

A very complex and somewhat dual role of autophagy has also been described in fibrotic changes in other organs, such as renal fibrosis. While autophagy induction during renal injury protects MC [40, 41], the disruption of ATG7 with siRNA or 3-MA treatment [4] but not with siBeclin1 [42], was shown to diminish collagen type 1 levels in cultured MC. These findings, as do ours, point to a tangled role of autophagy in the activation of fibroblasts.

This study supports the already existing idea that modulation of autophagy is a promising therapeutic target for the treatment of LF. Currently, liver transplant is the only curative therapy for LF due to the lack of specific antifibrotic drugs. In addition to interest in developing novel therapies, drug repurposing has been proposed given the advantage of already knowing the safety profile and bioavailability of existing drugs. The non-nucleoside reverse transcriptase inhibitor RPV is a widely used anti-HIV agent, which, in addition to its efficacy, possesses a better safety profile than other antiretroviral drugs, especially regarding hepatotoxicity and lipid metabolism [43–47]. The present study reaffirms RPV as a promising novel therapeutic avenue in the treatment of LF.

## Material and methods

### Reagents

Unless stated otherwise, chemicals were from Sigma-Aldrich. RPV (Sequoia Research Products) was dissolved in DMSO (stock 5 mM, employed at a final concentration of 1–8 μM), a solvent used as a control treatment (denoted as “vehicle”) and employed for all statistical comparisons. Rapamycin, wortmannin and TGF-β1 (Myltenyi Biotec) were also dissolved in DMSO, while 3-MA and CQ were prepared in water.

### Mouse model of chronic liver disease

Female C57BL/6 J 9-week-old mice (Janvier Labs) (20 ± 3 g of body weight) were randomly assigned to different experimentation groups (10 mice per group). We employed G*Power statistical software to calculate the sample size required for the in vivo experiments, based on the number of groups and the statistical analyses we planned to perform for each study, as well as on different input parameters obtained in previous in vivo experiments in liver samples: effect size = 0.38, alpha err prob = 0.05, power = 0.8. No blinding was applied in both in vivo experiments. To induce NAFLD, a nutritional model was employed for which animals received a normal diet (ND) or high-fat diet (HFD) (Ssniff Labs; EF R/M D12331 mod.*/Surwit; 59% fat and 2% free cholesterol) for 12 weeks. Both groups were subdivided into two groups, one co-administered with RPV and the other with the vehicle (Veh, DMSO). For the CCl_4_ model of hepatic injury, animals were randomly divided in 3 experimental groups: CCl_4_ (0.5 mg/kg (50 μL) in corn oil, intraperitoneal, three times per week), RPV and its Veh (DMSO), which were administered for 4 weeks. In both protocols of liver injury, RPV was administered orally, daily at 5 mg/kg/day (0.1 mg RPV in 10 μL DMSO). Animal dosage (oral) of RPV was calculated using the normalised interspecies allometric scaling factor established by the FDA to obtain the equivalent to of the maximum daily therapeutic dose of RPV (25 mg) [48]. All animals were given *ad libitum* access to water and chow. Mice were sacrificed using isoflurane and liver tissue was snap-frozen in liquid nitrogen and then stored at -80 ° C. All animal procedures were performed in accordance with the University of Valencia’s guidelines for the care and use of laboratory animals and were approved by the local ethics committee (2014/VSC/PEA/00188).

### Cell culture and treatment

LX-2 cells are a human immortalized HSC line which has been extensively characterized and retains key features of signaling and metabolism for which it is considered a suitable model of human hepatic fibrogenesis. It was purchased from Sigma-Aldrich and routinely cultured in DMEM with a high glucose concentration supplemented with 10% heat-inactivated FBS, 100 U/mL penicillin and 100 μg/mL streptomycin. Cell cultures were maintained in a cell culture incubator (MCO-19AICUV-PE, Panasonic Healthcare Co. Ltd.) at 37 °C, with a humidified 5% CO_2_/95% air atmosphere (AirLiquide Medical). Subculturing was performed using 0.25% Trypsin-EDTA and subconfluent cell cultures of passage number lower than 25 were used for all the experiments. Cells were treated with clinically relevant concentrations of RPV for 24–48 h. LX-2 were activated with the multipotent cytokine TGF-β (2.5 ng/mL), a well-known stimulator of HSC proliferation and fibrogenesis [14]. As a positive control of autophagy induction, we employed rapamycin (5 μM), which acts by inhibiting mammalian target of rapamycin (mTOR), resulting in the dephosphorylation of ULK1 and initiation of autophagy [10], while autophagy was pharmacologically inhibited with 3-MA, wortmannin or CQ. 3-MA (used at 2.5 mM) and wortmannin (used at 1 μM) act by blocking autophagosome formation via the inhibition of type III phosphatidylinositol 3-OH-kinases (PI3K) [34, 49, 50]. CQ (used at 60 μM) is a lysosomal lumen alkalizer that inhibits later stages of the autophagic process by inhibiting the fusion of the autophagosome with the lysosome [51]. Primary human HSC were purchased from Zenbio and cultured in DMEM with a high glucose concentration supplemented with 10% iFBS, 100 U/mL penicillin, 100 μg/mL streptomycin and 2.5 µg/mL amphotericin B, and used for experiments as subconfluent cell cultures of passage number lower than five. Cells were routinely tested for mycoplasma contamination.

### Transfection experiments

Transient silencing of *SQSTM1*, *ATG5* and *BECN1* was carried out by RNA interference. SignalSilence® unconjugated control siRNA (Cell Signaling Technology) was used as control. Transfection was performed using Lipofectamine™ 2000 transfection reagent (ThermoFisher Scientific), according to the manufacturer’s instructions. Cells were incubated for 5 h with serum-free OptiMEM (ThermoFisher Scientific) containing siRNA (1.5 nM in the case of si*SQSTM1*, from Santa Cruz Biotechnology, 100 nM for si*ATG5* from Dharmacon and 150 nM for si*BECN1*, from Santa Cruz Biotechnology), and 5 μL of LipofectamineTM 2000 per well for 6-well-plates. After 24 h, the specific treatments were performed.

### Cell viability assay

Cell viability was assessed by the acid phosphatase assay, which is based on the conversion of pNPP to p-nitrophenol by cytosolic acid phosphatase [52]. Cells (96-well-plate, 3 × 10^4^ cells/well) were treated with TGF-β alone or in the presence of RPV at increasing concentrations. In the case of the transfection experiments, transfection was performed the day before treatment. After 48h-treatment, the medium was removed, cells were washed with 100 μL of PBS, 100 μL of assay buffer (0.1 M sodium acetate at pH 5.0, 0.1% Triton X-100, and 7.25 mM p-nitrophenyl phosphate) was added per well and plates were then incubated at 37 °C for 2 h. The reaction was finally halted with the addition of 50 μL/well of NaOH 1 M and color development was assayed at 405 nm using a Multiskan® EX plate reader (Thermo Scientific). Non-enzymatic hydrolysis of the pNPP substrate was determined by adding assay buffer to the wells which did not contain cells. Treatment with the well-known inducer of apoptosis, staurosporine (STS, 0.1 μM) was employed as a positive control.

### RT-qPCR

RNA extraction from liver tissue was performed using TriPure Isolation Reagent (Roche Life Science). Liver samples (30-40 mg) were homogenized by MACS™ Dissociator (MACS Miltenyi Biotec) in 750 μL TriPure and centrifuged (16000 × *g*, 15 min, 4 °C). After 150 μL chloroform were added to the supernatant, samples were vigorously vortexed, incubated (ice, 15 min) and centrifuged (16000 g, 15 min, 4 °C). The colorless aqueous upper phase was transferred to new tubes in which RNA was precipitated by incubation with 500 μL isopropanol (o/n, –20 °C), pelleted by centrifugation (16000 × *g*, 20 min, 4 °C), washed with 1 mL 70% ethanol, pelleted again and resuspended in 50 μL RNase-free water. RNA isolation from cell culture was performed using the RNeasy® Mini Kit (Qiagen). Cell pellets were resuspended in 350 μL lysis buffer and RNA was eluted in 30 μL RNase-free water. The purity and concentration of the RNA were determined spectrophotometrically (NanoDrop™ ND-1000 spectrophotometer, Thermo Scientific). cDNA was synthetized employing the PrimeScript™ RT Reagent Kit (Perfect Real Time) (TaKaRa Bio Inc.) in a reaction with 2 μg RNA and 1X PrimeScript Buffer, 1 μL PrimeScript RT Enzyme Mix I, 50pmol Random 6-mers and 25 pmol Oligo dT Primer (20 μL final volume) in a GeneAmp® PCR System 2400 (PerkinElmer Inc., Waltham) under the following conditions: 37 °C for 15 min, 85 °C for 5 s and 4 °C until storage. qRT-PCR was performed with SYBR® Premix Ex Taq™ (Tli RNaseH Plus) (TaKaRa Bio Inc.) containing TaKaRa Ex Taq HS, dNTP mixture, Mg^2+^, Tli RNase H and SYBR Green I by mixing 1 μL cDNA, 5 μL SYBR® Premix Ex Taq™, 2 μM primers (forward and reverse) and RNase-free water (10 μL final volume) in a Lightcycler® 96 Real-Time PCR System (Roche Life Science) following this protocol: 95 °C for 30 s; 95 °C for 5 s; 60 °C for 20 s (50 cycles); 95 °C for 1 s; 65 °C for 15 s; 95 °C for 1 s and 40 °C for 30 s. All experiments were performed in duplicate, together with a negative control (RNase-free water). The primer pairs for mouse *Sqstm1* (sense 5ʹ-GGTTGCCTTTTCCAGTGACG-3ʹ and antisense 5ʹ-TCGCAGACGCTACACAAGTC-3ʹ) and *Actb* (sense 5ʹ -GCCAACCGTGAAAAGATGACC-3ʹ and antisense5ʹ-GAGGCATACAGGGACAGCAC-3ʹ) were synthetized by Metabion and Sigma-Aldrich, respectively. The primer pairs for human *SQSTM1* (sense 5ʹ-GGTTGCCTTTTCCAGTGACG-3ʹ and antisense 5ʹ-TCGCAGAGGCTACACAAGTC-3ʹ) and *GAPDH* (sense 5ʹ-CTTCTTTTGCGTCGCCAGCC-3ʹ and antisense5ʹ-TTCTCAGCCTTGACGGTGCC-3ʹ) were synthetized by Integrated DNA Technologies and Metabion, respectively. qRT-PCR data were analysed using the comparative CT method by which Fold Change = 2-Δ(ΔCT), where ΔCT = CT (target gene)—CT (housekeeping gene), and Δ(ΔCT) = ΔCT (treated)—ΔCT (control). *GAPDH* or *ACTB* was used as a housekeeping gene.

### Protein expression analysis by western blot

SDS-PAGE and protein transfer to nitrocellulose membranes were performed using standard techniques (Bio-Rad Laboratories). In order to isolate the whole-cell proteins, liver samples (20–35 mg) were homogenized in 900 μL extraction buffer (0.066 M Tris-HCl pH 7.5, 1 mM EGTA, 1 mM Na_3_VO_4_, 1 mM NaF and the protease inhibitor Complete Mini™ Protease Inhibitor Cocktail from Merck) using a MACS™ Dissociator (MACS Miltenyi Biotec). After adding 10 μL of 10% NP-40 Surfact-Amps™ (ThermoFisher Scientific), samples were sonicated (5 min, 15 °C) and centrifuged (16000 g, 40 min, 4 °C). For cell culture, cell pellets were lysed in 75-100 μL of PhosphoSafe™Extraction Reagent (EMD Millipore Corp.) supplemented with Complete Mini™ Protease Inhibitor Cocktail, vortexed (15 sec), incubated (5 min, RT) and centrifuged (4 °C, 5 min, 16000 rpm). In both cases, protein content was quantified by the BCA assay (Pierce™ BCA Protein Assay Kit, ThermoFisher Scientific). Primary antibodies included rabbit polyclonal—anti-GAPDH (1:15000, Sigma-Aldrich), anti-LC3 (1:1000, Sigma-Aldrich), anti-Beclin-1 (1:1000, Invitrogen), anti-ATG5 (1:1000, Cell Signaling) and anti-ATG7 (1:1000, Cell Signaling) and mouse monoclonal- anti-p62/SQSTM1 (1:1000, Santa Cruz Biotechnology). The secondary antibody were anti-rabbit (1:5000, Vector laboratories) and anti-mouse (1:2000, ThermoFisher Scientific). Immunolabeling was detected by enhanced chemiluminescence visualized with a digital luminescent image analyser (Fujifilm LAS-3000 Imager, Fujifilm), employing Luminata™ Crescendo Western HRP substrate (Merck), or SuperSignal™ West Femto Maximum Sensitivity Substrate (ThermoFisher Scientific). Densitometric analysis was performed using Multi Gauge V3.0 software (Fujifilm). Protein expression is represented as a percentage of control (average expression in the control group was considered 100%), using GAPDH as a loading control.

### Fluorescence microscopy

In all cases, cells were seeded (1.5 × 10^4^ /well) in a sterile µ-Slide 8 well chamber slide (Ibidi) and left to adhere over-night. Imaging was performed on inverted confocal laser-scanning microscope.

### Tandem fluorescence microscopy of eGFP/mCherry system

Autophagic flux can be monitored using a tandem fluorescence-tagged LC3 such as mCherry-GFP [53]. Unlike GFP, whose fluorescence is sensitive to the lysosomal acidic conditions, mCherrry maintains its structure under the same conditions. This implies that the expression of mCherry without GFP signal (net red fluorescence) and co-localization of GFP and RFP (net yellow fluorescence) indicate the presence of autolysosomes and autophagosomes, respectively. LC3- or p62-mCherry-GFP construct (kindly gifted by Prof. José M. Fuentes from the University of Extremadura, Spain) was delivered by transfection using lipofectamine 2000 (ThermoFisher Scientific) and cells were incubated for 4 hours. The following day, cells were treated with 1, 2, and 4 μM RPV and 2.5 ng/mL TGF-β or 5 μM rapamycin (positive control) for 24 h. Next, adherent cells were washed twice with Krebs-HEPES buffer (KB, 140 mM NaCl, 5.9 mM KCl, 1.2 mM MgCl2, 15 mM HEPES) and the medium was replaced with 300 μL/well of KB containing 5 mM D-glucose, 2.5 mM CaCl_2_ and 100 nM Hoechst 33342 (Sigma Aldrich), a nuclei-staining fluorescent dye. The plate was then transferred to a heated stage above a HCX PL APO CS 40.0 × 1.25 NA oil immersion UV objective lens on LeicaTCS SP2 microscope. Measurements were performed using lasers of 488, 543 and 361 nm for excitation of GFP, mCherry and Hoechst 33342, respectively, with a pinhole airy unit of 1 AU. Emission of Hoechst 33342, eGFP and mCherry was measured in the range of 400-470, 496-548 and 556-657 nm, respectively. Z-stacks were created by selecting a series of optical slices covering the entire length of the cell with a step size of 0.4 μm at a resolution of 512×512 pixels and a frame average of 4.

### Cyto-Id

The CYTO-ID® Autophagy Detection Kit 2.0 (Enzo Biochem Inc.) offers a rapid and quantitative approach to monitoring autophagy in live cells without the need for cell transfection. Cyto-Id selectively labels accumulated autophagic vacuoles including pre-autophagosomes, autophagosomes, and autolysosomes (autophagolysosomes) with minimal staining of lysosomes. After 48 h-treatment, cells were incubated with 100 µL/well of staining solution (2 µL of Cyto-Id in 1 mL of KB and 100 nM Hoechst 33342) for 30 min at 37 °C; after this, cells were washed with KB and the medium replaced with KB with 100 nM Hoechst, 5 mM D-glucose and 2.5 mM CaCl_2_. Cells were then transferred to a heated stage above a HCX PL APO CS 40.0 × 1.25 NA oil immersion UV objective lens on LeicaTCS SP2 microscope. Measurements were performed using lasers of 488 and 361 for excitation of Cyto-Id and Hoechst 33342, respectively, with a pinhole airy unit of 1 AU. Detection ranges were set to 495-693 and 427-487 nm for Cyto-Id and Hoechst respectively. For hHSC, cells were imaged on a heated stage of the inverted confocal laser-scanning microscope (LeicaTCS SP8) above a HC PL APO CS2 40.0 × 1.30 NA oil immersion objective lens equipped with a white laser (WLL) and diode laser. Samples were excited at 488 and 405 nm for Cyto-Id and Hoechst, respectively.

### Assessment of the lysosomal content using Lysotracker Red

After 48 h-treatment, cells were stained with 100 nM LysoTracker™ Red DND-99 (LTR, ThermoFisher) to detect the presence of lysosomes and 100 nM of Hoechst 33342 per well for 30 min at 37 °C. Subsequently, the medium was removed, cells were washed with KB and incubated with KB containing 5 mM D-glucose, 2.5 mM CaCl_2_ and 100 nM Hoechst 33342. The plate was transferred to a heated stage above a HCX PL APO CS 40.0 ×1.25 NA oil immersion UV objective lens on LeicaTCS SP2 microscope. Lasers of 361 and 543 for excitation of Hoechst 33342 and LTR, respectively were used, with a resolution of 2.7 pixel/ μm and a pinhole airy unit of 1 AU. Detection ranges were set to 400-470 nm and 568-666 nm for Hoechst 33342 and LTR, respectively.

### Image analysis

Images were first processed with ImageJ2 (National Institute of Health, Bethesda, MD, USA). Background was subtracted from stacks using the “subtract background” function with the sliding paraboloid option set to a rolling ball radius of 50 pixels. Images were analysed using a customised processing pipeline with CellProfiler r4.0.6 (Broad Institute, USA). For the tandem fluorescence images, individual cell stacks that were positive for the construct expression were individually cropped and after background subtraction, red and green punctae were identified with the identify primary objects module with a typical diameter of objects set between 2 and 5 pixels and a global threshold strategy with the Otsu method. Green objects were then related to red punctae using the relate objects module and classified to obtain the number of red, green and double positive (red and green) punctae. For LTR and Cyto-Id, a correct illumination module was applied with a smoothing Gaussian method, nuclei were determined by employing the identify primary objects module with a typical diameter of objects set between 40 and 80 pixels and a global threshold strategy with the Otsu method. For the punctae signal, we once again employed a correct illumination module followed by an enhance features module (feature type set to speckles). Punctae were identified by employing the identify primary objects module with a typical diameter of objects set between 2 and 10 pixels and a global threshold strategy with the Otsu method. Finally, the set of punctae identified was related to its own nucleus with the expand objects and relate objects modules.

### Presentation of data and statistical analysis

All images are representative of at least 3 independent experiments. In most cases, data are represented as % of control, with untreated cells considered 100%. Data shown as median ± IQR or mean ± SEM were analysed using GraphPad Prism V.8.01 (GraphPad Software, San Diego, CA, USA) with unpaired or paired Student’s *t*-test (#*p* < 0.05) or a one-way analysis of variance (ANOVA) followed by a Bonferroni test (*p < 0.05).

## Supplementary information

Supplementary Figures

Reproducibility checklist

## Data Availability

All data describing the findings of this study are available within this article and supplementary information.
