# Supplementary material for: Implication of autophagy in the antifibrogenic effect of Rilpivirine: when more is less
Source: Cell Death Dis. 2022 Apr 20;13(4):385. doi: 10.1038/s41419-022-04789-7 (PMC9021290; doi:10.1038/s41419-022-04789-7)
Supplement: Supplementary file 1 — Supplementary Figures [file 41419_2022_4789_MOESM1_ESM.docx]

**Supplementary material to:**

**Implication of autophagy in the antifibrogenic effect of Rilpivirine: when more is less**

Federico Lucantoni, Ana M. Benedicto, Aleksandra Gruevska, Ángela B. Moragrega, Isabel Fuster-Martínez, Juan V. Esplugues, Ana Blas-García and Nadezda Apostolova

**Supplementary figure 1. mRNA levels of several profibrotic markers in whole-liver samples of a diet model of chronic liver injury**. Female C57BL/6J mice received normal diet (ND) or a high-fat diet (HFD) for 12 weeks, and were orally administered (p.o.) either RPV (5 mg/kg/day) or its vehicle (Veh, DMSO). Relative gene expression of transforming growth factor beta (*Tgfb*), vimentin (*Vim*) and alpha smooth muscle actin (*Acta2*) were determined by RT-qPCR and expression was normalized with that of GAPDH (mRNA levels in the Veh-ND group were considered 100%). Data represented as mean ± SEM of 8 mice were statistically analysed by Student´s *t*-test of RPV treatment vs Veh (** represents *p* value < 0.01 and *** represents *p* value < 0.001) and HFD vs ND (# represents *p* value < 0.05 and ## represents *p* value < 0.01).

**Supplementary figure 2. Effect of 3-MA or silencing of main autophagic genes on LX-2 viability**. LX-2 cells were treated with 3-MA, wortmannin or with *ATG5*, *BECN1 or* *SQSTM*1 siRNA for 48h and cell viability assessed with acid phosphatase assay. Data represent mean ± SEM (n=3-5) and are expressed in relation to those of untreated cells in each experiment, which was considered 100%. Statistical analysis was performed with unpaired Student´s *t*-test vs vehicle (in the case of 3-MA) or vs control siRNA (in the case of the RNA interference experiments); * represents a *p* value < 0.05.
